# O uso do balão com paclitaxel no tratamento da reestenose intra-stent no segmento fêmoro-poplíteo

**DOI:** 10.1590/1677-5449.010616

**Published:** 2017

**Authors:** Julio Cesar Peclat de Oliveira, Ana Paula Rolim Maia Peclat, Fernando Tebet Ramos Barreto, João Marcos Fonseca, Diogo Di Batista de Abreu e Souza, Stenio Karlos Alvim Fiorelli, Rossano Kepler Alvim Fiorelli, Marcos Arêas Marques

**Affiliations:** 1 Clínica Julio Peclat, Departamento de Cirurgia Vascular, Rio de Janeiro, RJ, Brasil.; 2 Universidade Federal do Estado do Rio de Janeiro – UNIRIO, Hospital Universitário Gaffrée e Guinle, Rio de Janeiro, RJ, Brasil.; 3 Universidade do Estado do Rio de Janeiro – UERJ, Hospital Universitário Pedro Ernesto – HUPE, Rio de Janeiro, RJ, Brasil.

**Keywords:** doença arterial periférica, angioplastia, reestenose de enxerto vascular

## Abstract

**Contexto:**

A reestenose intra-stent por hiperplasia miointimal pós-angioplastia é uma intercorrência frequente e que limita a perviedade do procedimento a longo prazo. A terapia com balões revestidos de droga com ação antiproliferativa pode ser uma alternativa no tratamento dessa complicação.

**Objetivos:**

Demonstrar eficácia e as complicações (óbito, grandes amputações, etc.) do balão farmacológico no tratamento da reestenose intra-stent de segmento femoropoplíteo.

**Métodos:**

Estudo de coorte retrospectivo de 32 pacientes consecutivos tratados entre os anos de 2012 e 2016, submetidos a terapia de reestenose intra-stent de segmento femoropoplíteo com angioplastia com balão farmacológico revestido com paclitaxel. A taxa de sucesso foi mensurada pela ocorrência de sucesso do procedimento e reestenose inferior a 50% em avaliação por eco-Doppler colorido 30, 90 e 180 dias após o procedimento.

**Resultados:**

Quatro pacientes (12,5%) apresentaram reestenose superior a 50%, sendo um (3,1%) após 90 dias e três (9,4%) após 180 dias, conferindo uma taxa de sucesso de 87,5% ao procedimento. Após 180 dias, todos os pacientes referiam melhora ou cessação dos sinais e/ou sintomas apresentados antes do procedimento. Não houve óbitos, e complicações ocorreram apenas em dois casos, no pós-operatório imediato.

**Conclusões:**

Os resultados a curto prazo da terapia com balão farmacológico são promissores, com redução na taxa de reestenose e baixo índice de complicações. Ainda precisam ser apresentados estudos demonstrando os efeitos a longo prazo dessa terapia, assim como seu impacto econômico quando comparada a outros procedimentos.

## INTRODUÇÃO

**Figura 1:**
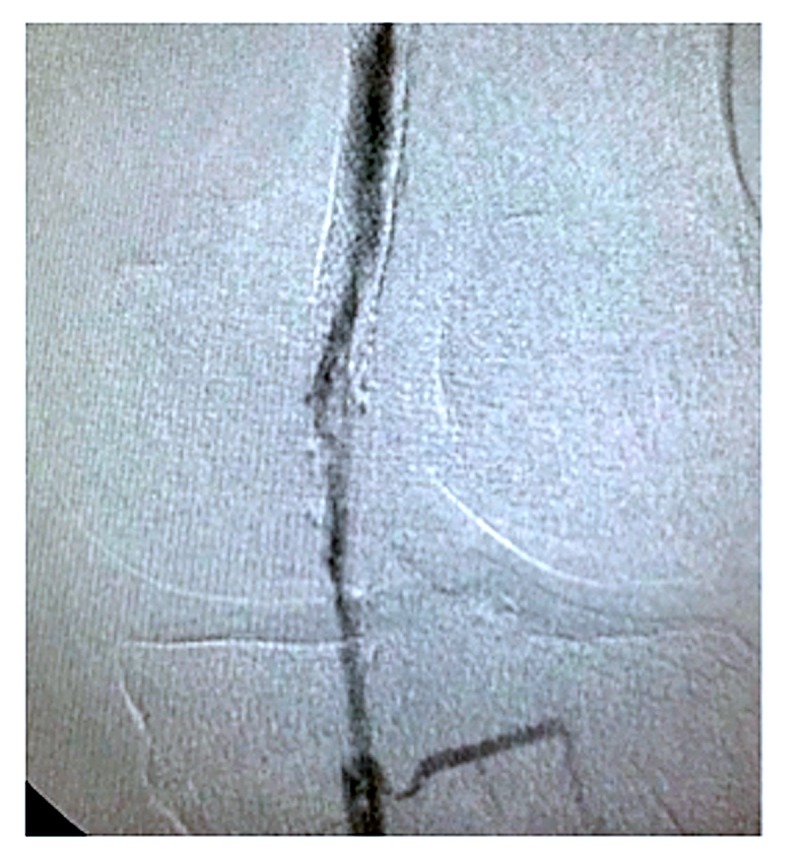
Arteriografia pré-tratamento evidenciando reestenose intra-stent de aproximadamente 85% no segmento mais crítico, em transição femoropoplítea.

O comprometimento da perfusão sanguínea dos membros inferiores na doença arterial periférica (DAP) constitui um evento de morbidade potencialmente elevada. Apesar do sucesso incialmente obtido pela angioplastia transluminal percutânea por balão (ATPB), a taxa de reestenose pode chegar a 60% em 1 ano^1^. O uso de stents convencionais (SC) apareceu como uma tentativa de reduzir essa complicação, com algum sucesso. No entanto, ainda se observa uma taxa de reestenose intra-stent (RIS) relativamente alta, entre 18 e 37%, em 1 ano após o tratamento com SC em segmento femoropoplíteo (Figura 1)^2^.

Alternativas para o tratamento da RIS incluem a ATPB e o implante de novo SC, com resultados pouco satisfatórios, e o desenvolvimento de novas tecnologias, como a angioplastia com balões revestidos com drogas (BRD), mais notoriamente com paclitaxel, que apresenta efeito antiproliferativo na parede arterial.

O objetivo do estudo é avaliar a eficácia do uso dos BRD como opção de tratamento para a RIS, além da ocorrência de complicações decorrentes desse uso.

## MÉTODOS

Foi realizada análise de coorte retrospectiva de prontuários de pacientes submetidos a angioplastia transluminal percutânea de segmento femoropoplíteo entre 2012 e 2016. Foram selecionados 32 pacientes (19 do sexo masculino e 13 do sexo feminino) com idade entre 56 e 77 anos (média de 66,5 anos) que se adequavam aos critérios de inclusão propostos neste estudo: RIS superior a 50% (velocidade de pico sistólico > 180 cm/s) há mais de 3 meses, claudicação leve a lesão tecidual pequena (Rutherford 2-5), pelo menos uma artéria de deságue até o pé e extensão da lesão menor que 27 cm. Os critérios de exclusão (todos utilizados no COPA CABANA Trial^3^) foram: mais de duas lesões simultâneas, fratura do stent, insucesso na recanalização da lesão, trombose aguda e lesão proximal sem tratamento prévio, além da participação do paciente em outros estudos similares.

O intervalo entre o procedimento primário e o diagnóstico e tratamento da RIS variou entre 6 meses e 5 anos. Vinte e quatro pacientes (75%) foram tratados com o BRD IN.PACT™ Admiral® (Medtronic®) e oito (25%), com o BRD Lutonix® (Bard®).

O sucesso técnico foi definido como estenose residual inferior a 30% da lesão alvo quando comparadas arteriografias intraoperatórias pré- e pós-angioplastia. Estenoses superiores a 30%, trombose aguda, embolia distal e dissecção foram considerados critérios para o insucesso técnico.

O procedimento foi considerado satisfatório quando se observou sucesso técnico e um índice de reestenose inferior a 50%, quando comparado o eco-Doppler colorido (EDC) pré-operatório aos realizados 30, 90 e 180 dias após a intervenção, como é a rotina do serviço, pelo mesmo profissional que realizou o procedimento no momento do diagnóstico de RIS. Foram avaliados também os sinais e sintomas (presença e intensidade da claudicação e dor em repouso, presença ou não de lesões tróficas de características isquêmicas em membros inferiores) demonstrados pelos pacientes, assim como as complicações apresentadas.

Não houve exclusões de participantes por perda de seguimento ou falta das informações necessárias ao estudo.

O trabalho obteve aprovação do Comitê de Ética em Pesquisa.

## RESULTADOS

A taxa de mortalidade do procedimento foi zero. O tempo de internação após o procedimento variou de 1 a 4 dias e ocorreram complicações no pós-operatório imediato em dois pacientes (hematoma não cirúrgico no sítio de punção e elevação de escórias nitrogenadas).

Após 6 meses de acompanhamento, todos os 32 pacientes referiram melhora ou cessação dos sintomas apresentados antes da intervenção com o BRD, além de experimentarem aumento das distâncias percorridas sem claudicação, cicatrização de lesões tróficas e ausência de dor em repouso nos membros inferiores (Tabela 1). Não foi necessária nenhuma amputação de grande porte (acima do tornozelo).

**Tabela 1:** Modificação da sintomatologia apresentada ao longo do acompanhamento, seguindo os critérios de Rutherford (0 = condição pré-operatória).

	**Tempo**			
**Rutherford**	**0**	**30**	**90**	**180**
**0**	- (0%)	8 (25%)	11 (34,4%)	16 (50%)
**1**	- (0%)	11 (34,4%)	15 (46,9%)	13 (40,6%)
**2**	7 (21,8%)	10 (31,2%)	4 (12,5%)	3 (9,4%)
**3**	12 (37,5%)	- (0%)	- (0%)	- (0%)
**4**	8 (25%)	- (0%)	- (0%)	- (0%)
**5**	5 (15,7%)	3 (9,4%)	2 (6,2%)	- (0%)
**6**	- (0%)	- (0%)	- (0%)	- (0%)
**Total**	32 (110%)	32 (110%)	32 (110%)	32 (110%)

**Figura 2:**
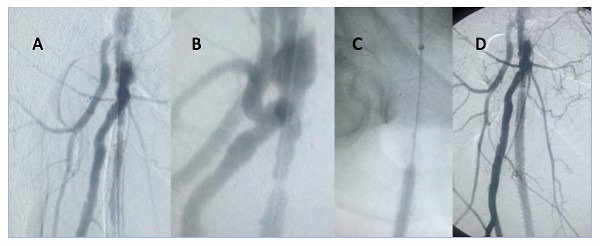
Angioplastia por estenose intra-stent em um dos casos do estudo. (A) estenose extensa intra-stent; (B) imagem em zoom de área de oclusão na porção proximal do stent; (C) angioplastia com balão farmacológico; (D) controle final, com melhora das lesões prévias.

Ao fim do acompanhamento, quatro pacientes (12,5%) apresentaram reestenose superior a 50%, sendo um (3,1%) aos 90 dias e três (9,4%) aos 180 dias após o EDC arterial, conferindo uma taxa de sucesso de 87,5% ao procedimento (Tabela 2 e Figura 2).

**Tabela 2:** Resultados referentes ao seguimento com 30, 90 e 180 dias após tratamento de reestenose intra-stent por avaliação com eco-Doppler colorido.

**Resultados**			
	**30 dias**	**90 dias**	**180 dias**
**Sucesso**	32 (100%)	31 (96,9%)	28 (87,5%)
**Insucesso**	0 (0%)	1 (3,1%)	4 (12,5%)

## DISCUSSÃO

A superioridade dos BRD em relação aos SC já foi demonstrada em diversos estudos sobre angioplastia da artéria femoral superficial, com melhor perviedade primária e redução das intervenções em complicações^4^-^6^. Além da eficácia em termos de melhora dos sintomas, tempo livre de reintervenção e redução na taxa de amputações, foi demonstrada segurança similar na realização do procedimento, o que pode ser explicado pela semelhança da técnica entre os procedimentos.

Os resultados obtidos em nossa casuística são compatíveis com os principais estudos publicados sobre o tratamento da RIS com BRD^3^,^7^. No entanto, ainda são poucos os estudos sobre o tema, e estes não apresentam resultados a longo prazo. No momento, existem alguns estudos em andamento investigando a eficácia dos BRD na RIS da artéria femoral superficial, se destacando o PLAISIR Study, o ISAR-PEBIS RCT e o PACUBA I RCT^3^.

Outro ponto a ser considerado é a relação custo-benefício dos BRD se comparados à ATPB e aos SC. Há estudos sugerindo que os BRD são superiores^8^.

Por se tratar de um estudo retrospectivo de curto prazo, com número limitado de pacientes, com alguns vieses (como acompanhamento por mais de um ultrassonografista ou variados tipos de stents), com avaliação não cega, sem adequada avaliação da qualidade de vida, dos custos e do impacto econômico, entendemos que serão necessários estudos prospectivos que nos permitam avaliar se o resultado superior a curto prazo pode ser reproduzido a médio e longo prazo, assim como a relação custo-benefício do BRD quando comparado a outros tipos de intervenção.

## CONCLUSÃO

A terapia da RIS com balão revestido com paclitaxel é factível e pode ser realizada com segurança (baixa incidência de complicações relativas ao método; mortalidade nula), demonstrando excelentes resultados a curto prazo.
