# Supplementary material for: O uso do balão com paclitaxel no tratamento da reestenose intra-stent no segmento fêmoro-poplíteo
Source: J Vasc Bras. 2017 Jan-Mar;16(1):31–4. [Article in Portuguese] doi: 10.1590/1677-5449.010616 (PMC5829689; doi:10.1590/1677-5449.010616)
Supplement: Translation

# Translation: Use of paclitaxel-coated balloon to treat in-stent restenosis in femoropopliteal segments

**Authors:** Julio Cesar Peclat de Oliveira, Ana Paula Rolim Maia Peclat, Fernando Tebet Ramos Barreto, João Marcos Fonseca, Diogo Di Batista de Abreu e Souza, Stenio Karlos Alvim Fiorelli, Rossano Kepler Alvim Fiorelli, Marcos Arêas Marques

**Affiliations:** 1 Clínica Julio Peclat, Departamento de Cirurgia Vascular, Rio de Janeiro, RJ, Brazil.; 2 Universidade Federal do Estado do Rio de Janeiro – UNIRIO, Hospital Universitário Gaffrée e Guinle, Rio de Janeiro, RJ, Brazil.; 3 Universidade do Estado do Rio de Janeiro – UERJ, Hospital Universitário Pedro Ernesto – HUPE, Rio de Janeiro, RJ, Brazil.

**Keywords:** peripheral artery disease, balloon angioplasty, vascular graft restenosis

## Abstract

**Background:**

In-stent restenosis due to myointimal hyperplasia after angioplasty is common and limits long-term patency. Treatments using balloons coated with antiproliferative drugs may offer an alternative option for this pathology.

**Objectives:**

To demonstrate the efficacy and complications (death, major amputations, etc.) of drug-coated balloons for treatment of in-stent restenosis in femoropopliteal segments.

**Methods:**

This was a retrospective cohort study of 32 consecutive patients treated between 2012 and 2016 who underwent treatment to correct in-stent restenosis in the femoropopliteal segment using paclitaxel-coated balloons. The success rate was measured in terms of technical success and restenosis of less than 50% on Doppler ultrasonography at 30, 90, and 180 days after the procedure.

**Results:**

Four patients (12.5%) exhibited restenosis greater than 50%, one (3.1%) after 90 days and three (9.4%) after 180 days, equating to a success rate of 87.5% of procedures, and by 180 days all patients experienced improvement or cessation of the signs and/or symptoms they had presented prior to the procedure. There were no deaths and complications occurred in just 2 cases in the immediate postoperative period.

**Conclusions:**

Short-term results are promising, with reductions in the magnitude of restenosis and a low rate of complications. Further studies are needed that can demonstrate the long-term effects and the economic impacts in comparison to other procedures.

## INTRODUCTION

Compromise to blood perfusion of the lower limbs in peripheral arterial disease (PAD) is an event of elevated potential morbidity. Although initial success rates after percutaneous transluminal balloon angioplasty (PTBA), restenosis rates can be as high as 60% within 1 year.^1^ Conventional stents (CS) have been used to attempt to reduce rates of this complication, with a certain degree of success. However, rates of in-stent restenosis (ISR) are still relatively high, at 18 to 37% at 1 year after treatment with CS in femoropopliteal segments (Figure 1).^2^

**Figure 1:** Pretreatment arteriography showing in-stent restenosis of approximately 85% at the most critical point, at the femoropopliteal transition.

Treatment options for ISR include PTBA and placement of a new CS, with fairly unsatisfactory results, and newly developed technologies, such as drug-coated balloons (DCBs), of which the most well-known is coated with paclitaxel, which has antiproliferative effects on the artery wall.

The objectives of this study are to investigate the efficacy of using DCBs as a treatment option for ISR, and to analyze the occurrence of complications related to their use.

## METHODS

A retrospective analysis was conducted of the medical records of a cohort of patients who had been treated with percutaneous transluminal angioplasty of femoropopliteal segments between 2012 and 2016. The cohort comprised 32 patients (19 males and 13 females) aged from 56 to 77 years (mean: 66.5 years) who met the inclusion criteria proposed for the study: ISR greater than 50% (peak systolic velocity > 180 cm/s) for more than 3 months, mild claudication, and minor tissue damage (Rutherford 2-5), at least one artery draining as far as the foot, and lesion length of less than 27 cm. The exclusion criteria (all of those used for the COPA CABANA Trial^3^) were as follows: more than two simultaneous lesions, stent fracture, failure to recanalize the lesion, acute thrombosis, and proximal lesion with no prior treatment, in addition to participation of patient in other similar studies.

Intervals between the primary procedure and diagnosis and treatment of ISR ranged from 6 months to 5 years. Twenty-four patients (75%) were treated with an IN.PACT™ Admiral® DCB (Medtronic®) and eight (25%), with a Lutonix® DCB (Bard®).

Technical success was defined as residual stenosis of the target lesion of less than 30%, based on comparison of pre-angioplasty and post-angioplasty intraoperative arteriographies. Greater than 30% stenosis, acute thrombosis, distal embolism, and dissection were all defined as technical failure.

The procedure was considered satisfactory when technical success was achieved and observed restenosis was less than 50%, based on comparison of preoperative color Doppler ultrasonography findings of examinations conducted with the same technique at 30, 90, and 180 days by the same professional who had conducted the examination in which ISR was detected, in accordance with routine procedure at the service. Patients’ signs and symptoms (presence and intensity of claudication and of pain at rest, presence or absence of trophic ulcers with ischemic characteristics on the lower limbs) and any complications suffered were also analyzed.

None of the study participants were excluded because of loss to follow-up or because any of the information needed for the study was missing.

This study was granted Research Ethics Committee approval.

## RESULTS

The mortality rate of this procedure was zero. The length of postoperative hospital stay varied from 1 to 4 days and there were complications during the immediate postoperative period in two patients (non-surgical hematoma at the puncture site and elevated nitrogen retention).

At 6-month follow-up, all 32 patients reported that the symptoms that they had experienced before intervention with the DCB had either improved or disappeared and also reported increases in the distances they were able to walk without claudication, healing of trophic ulcers, and absence of lower limb pain at rest (Table 1). No major amputations (above the ankle) were necessary.

**Table 1:** Changes to symptoms during follow-up, graded according to the Rutherford criteria (0 = preoperative condition).

	**Time (days)**			
**Rutherford**	**0**	**30**	**90**	**180**
**0**	- (0%)	8 (25%)	11 (34.4%)	16 (50%)
**1**	- (0%)	11 (34.4%)	15 (46.9%)	13 (40.6%)
**2**	7 (21.8%)	10 (31.2%)	4 (12.5%)	3 (9.4%)
**3**	12 (37.5%)	- (0%)	- (0%)	- (0%)
**4**	8 (25%)	- (0%)	- (0%)	- (0%)
**5**	5 (15.7%)	3 (9.4%)	2 (6.2%)	- (0%)
**6**	- (0%)	- (0%)	- (0%)	- (0%)
**Total**	32 (110%)	32 (110%)	32 (110%)	32 (110%)

By the final follow-up appointment, four patients (12.5%) exhibited restenosis greater than 50%, one (3.1%) at 90 days and three (9.4%) at 180 days after the arterial color Doppler ultrasonography, equating to a success rate of 87.5% of procedures (Table 2 and Figure 2).

**Table 2:** Results at 30, 90, and 180 days follow-up after in treatment of in-stent restenosis, assessed with color Doppler ultrasound.

**RESULTS**			
	**30 days**	**90 days**	**180 days**
**Success**	32 (100%)	31 (96.9%)	28 (87.5%)
**Failure**	0 (0%)	1 (3.1%)	4 (12.5%)

**Figure 2:** Angioplasty for in-stent stenosis in one of the cases in the study sample. (A) extensive in-stent stenosis; (B) zoomed image of the area of occlusion in the proximal portion of the stent; (C) pharmacological balloon angioplasty; (D) final control image, with improvement to earlier lesions.

## DISCUSSION

The superiority of DCBs in comparison to CS has already been shown in several studies investigating angioplasty of the superficial femoral artery, revealing better primary patency and reductions in rates of interventions because of complications.^4^-^6^ In addition to improved efficacy in terms of improvement of symptoms, time free from reintervention, and reductions in amputation rates, studies have also shown that the procedures have similar levels of safety, which is understandable considering the similar techniques employed.

The results achieved in our sample of patients are compatible with those reported in published studies of ISR treatment using DCBs.^3^,^7^ However, so far there have been few studies investigating this subject and those that exist do not report long-term results.

There are currently a number of ongoing studies investigating the efficacy of DCBs for ISR in the superficial femoral artery, among which the PLAISIR Study, the ISAR-PEBIS RCT, and the PACUBA I RCT are of note.^3^

Another point of interest is the cost-benefit relationship offered by DCBs in comparison to PTBA and CS. There are studies that suggest that DCBs are better in this respect.^8^

This was a short-term retrospective study of a limited number of patients, in which some possible sources of bias, such as follow-up with more than one sonographer, varied types of stents, were not assessed blind, insufficient evaluation of quality of life, costs, and economic impact. Therefore, we are aware that prospective studies are needed to enable assessment of whether the superior short-term results can be reproduced over the medium and long term, and also to compare the cost-benefit profile of DCBs to those of other types of intervention.

## CONCLUSIONS

Treatment of ISR with paclitaxel-coated balloons is feasible, it can be performed safely (the incidence of complications related to the method was low and mortality was zero), and it demonstrates excellent short-term results.
